# Letter to the Editor

**DOI:** 10.1155/S1064744996000609

**Published:** 1996

**Authors:** Pål Wölner-Hanssen

**Affiliations:** Department of Obstetrics and Gynecology University of Lund Lund Sweden


Infectious Diseases in Obstetrics and Gynecology 4:310 (1996)
© 1997 Wiley-Liss, Inc.

### Letter to the Editor

To the Editor:

In an abstract for the 23rd Annual Meeting of the Infectious Diseases Society for Obstetrics and Gynecology in Beaver Creek, CO, August 1996, we presented results from cultures between the fetal membranes after term deliveries.[1] The data showed bacterial growth in almost 70% of the 312 membranes. Doubts about the credibility of these data were put forward after the presentation. It was suggested that the specimens had been contaminated by non-sterile gloves used by the nurse-midwives obtaining the specimens. To solve this issue, we have performed the following controlled study. Culture specimens were obtained between the membranes from 17 term women delivering during daytime. We asked the nurse-midwives to obtain the specimens in the same way as they had done during the first study. Thereafter, one of the coauthors of the abstract (A.H.), using sterile gloves, split the membranes to approximately 2 in. from the membrane edge on the opposite side of the placenta. A nurse's aid streaked a charcoal-treated cotton-tipped swab between the membranes at their approximation and put it into Stuart's medium. The two specimens from each case were immediately taken to the microbiology laboratory for processing. The microbiologist was blinded as to who had obtained the specimens. The nurse-midwives were found to have handled the specimens appropriately, apart from using the same gloves as they had used to deliver the baby. However, the specimens obtained by the nurse-midwives were culture positive in 9 (53%), and those obtained by A.H. were positive in 2 (11%) of the 17 cases ($P = 0.02$, McNemar's test). Of the 9 culture-positive membrane cultures obtained by nurse-midwives, 6 showed growth of pathogens (1 *Escherichia coli,* 2 *Prevotella melaninogenicus,* 2 *Bacteroides fragilis,* and 1 *Peptostreptococci micros* plus *Prevotella buccae*). The bacteria isolated from specimens obtained by A.H. were lactobacilli in one case and coagulase-negative staphylococci in the other. In conclusion, it seems confirmed that the high rate of culture positivity reported in the abstract in fact reflects contamination from the gloves of the nurse-midwives.

*Pål Wölner-Hanssen, M.D., Ph.D.*

*Department of Obstetrics and Gynecology*

*University of Lund*

*Lund, Sweden*

### REFERENCE

1. Wölner-Hanssen P, Morsing E, Hägerstrand I, Holst E, Ljung Å, Herbst A: Fetal membrane microbial flora at term: No association with chorioamnionitis. In: Abstracts From the Infectious Diseases Society for Obstetrics and Gynecology, Annual Meeting, Beaver Creek, CO, August 14–17, 1996.

